# Cadmium-Induced Upregulation of Lipid Peroxidation and Reactive Oxygen Species Caused Physiological, Biochemical, and Ultrastructural Changes in Upland Cotton Seedlings

**DOI:** 10.1155/2013/374063

**Published:** 2013-12-26

**Authors:** Muhammad Daud Khan, Lei Mei, Basharat Ali, Yue Chen, Xin Cheng, S. J. Zhu

**Affiliations:** ^1^Institute of Crop Science, College of Agriculture and Biotechnology, Zhejiang University, Zijingang Campus, Hangzhou, China; ^2^Department of Biotechnology and Genetic Engineering, Kohat University of Science and Technology, Kohat 26000, Pakistan

## Abstract

Cadmium (Cd) toxicity was investigated in cotton cultivar (ZMS-49) using physiological, ultrastructural, and biochemical parameters. Biomass-based tolerance index decreased, and water contents increased at 500 *μ*M Cd. Photosynthetic efficiency determined by chlorophyll fluorescence and photosynthetic pigments declined under Cd stress. Cd contents were more in roots than shoots. A significant decrease in nutrient levels was found in roots and stem. A significant decrease in nutrient levels was found in roots and stems. In response to Cd stress, more MDA and ROS contents were produced in leaves than in other parts of the seedlings. Total soluble proteins were reduced in all parts except in roots at 500 *μ*M Cd. Oxidative metabolism was higher in leaves than aerial parts of the plant. There were insignificant alterations in roots and leaves ultrastructures such as a little increase in nucleoli, vacuoles, starch granules, and plastoglobuli in Cd-imposed stressful conditions. Scanning micrographs at 500 *μ*M Cd showed a reduced number of stomata as well as near absence of closed stomata. Cd depositions were located in cell wall, vacuoles, and intracellular spaces using TEM-EDX technology. Upregulation of oxidative metabolism, less ultrastructural modification, and Cd deposition in dead parts of cells show that ZMS-49 has genetic potential to resist Cd stress, which need to be explored.

## 1. Introduction

Cadmium (Cd) like other heavy metals such as arsenic, lead, and chromium is a persistent inorganic toxic pollutant, which comes mainly through various anthropogenic activities such as industrialization and mining [1]. It can be readily taken up by plant roots because of its relatively high mobility in the soil-plant system [2] and can pose serious threats to human health by entering into the food chain. Its presence in the environment poses several problems for both plants and animals at various functional levels. Plants are more prone to Cd stress than animals and experience various physiological, ultrastructural, and biochemical disturbances upon exposure to Cd. Physiological retardations such as limited water and nutrients' transportation, reduced mitochondrial respiration, low production of photosynthates, stunted growth, and reproduction have been observed due to Cd stress in plants [3]. Ultrastructural anomalies in plants like increase in number of nucleoli and vacuoles [4], condensed cytoplasm, reduced mitochondrial cristae, severe plasmolysis, highly condensed chromatin materials, enlarged vacuoles, disorganized chloroplastic structure and disrupted nuclear envelope [5], disorganized granal and stromal thylakoids and appearance of enlarged plastoglobuli in chloroplasts, and dilated thylakoid membranes have also been reported [6].

In Cd stressed conditions, the ultrastructural studies reveal the appearance of electron dense precipitates, which need to be analyzed for their chemical composition. Their analyses as well as distribution in cellular compartments are important to better comprehend the tolerance mechanisms in plant species [7]. This can be performed with various analytical technologies, such as energy-dispersive X-ray analyses (EDX) and electron energy loss spectroscopy (EELS), which are equipped with transmission and scanning electron microscopes. These are useful tools for studying subcellular distribution, compartmentalization, and speciation of heavy metals [8] and can precisely localize different heavy elements in the cellular compartments [7].

Cd stress can also inhibit various metabolic events in plants. Resultantly, cellular energy deficiency and oxidative stress are accelerated [9], which lead to the increased production of various free radicals and reactive oxygen species (ROS). They are such as the superoxide (O_2_^•−^), hydrogen peroxide (H_2_O_2_), and hydroxyl (OH^•^) radicals. They can directly damage the cells through peroxidation of poly unsaturated fatty acid of lipid membranes [10], protein oxidation, and DNA damage [3] and cause oxidative stress in cells. To avoid or to minimize the stressful effects of these radicals, various mechanisms get activated. For example, they either lower down Cd absorption and uptake, bind and sequester biomolecules, or synthesize antioxidant molecules [10, 11].

Antioxidant molecules are composed of various ROS-scavenging enzymatic and nonenzymatic antioxidants [3]. They are, for example, superoxide dismutase (SOD), peroxidase (POD), catalase (CAT), ascorbate peroxidase (APX), glutathione reductase (GR), and so forth. SOD catalyzes the dismutation of O_2_^•−^ to H_2_O_2_. CAT can dismutate H_2_O_2_ to oxygen and water, and APX reduces H_2_O_2_ to water by utilizing ascorbate as specific electron donor. Nonenzymatic antioxidant ROS detoxification mechanisms are mainly composed of ascorbate and glutathione (reduced and oxidized), as well as vitamins, flavonoids, alkaloids, and carotenoids [12].

Phytoremediation is a promising plant-based remediation of contaminated soils. Woody species such as willow and poplar have been widely exploited in recent years due to their resistance and accumulation potential of various metals [13]. Cotton is a woody perennial tree, which is widely grown as fiber and oil crop. It can tolerate various abiotic and biotic stress factors. The present study was designed to investigate responses of cotton seedlings to Cd short-term stress. They were subjected to various physiological, biochemical, and ultrastructural modifications and localization studies.

## 2. Materials and Methods

### 2.1. Plant Culture Conditions

An upland cotton cultivar (ZMS-49) was used in the present experiment. Uniform-sized seeds were surface sterilized using 70% ethanol for 3 min and then in 0.1% HgCl_2_ for 8–10 min. After several times washing with ddH_2_O, seeds were soaked overnight in dH_2_O. Next day, they were sown in a mixture of peat and vermiculite (7 : 3, v : v) for ten days under controlled growth chamber conditions. Seeds were kept in complete dark conditions for the first three days and thereafter a 16 h photoperiod of 50 *μ*molm^−2^s^−1^ under white fluorescent light was provided for for the next 7 days at a temperature of 28 ± 2°C culture temperature and 60% relative humidity. At the end of 10-day growth period, uniform seedlings were transferred to modified Hoagland solution for four hours acclimatization period. Hoagland media were composed of 500 *μ*M (NH_4_)_2_SO_4_, 500 *μ*M MgSO_4_, 200 *μ*M K_2_SO_4_, 1000 *μ*M KNO_3_, 600 *μ*M Ca(NO_3_)_2_·4 H_2_O, 200 *μ*M KH_2_PO_4_, 100 *μ*M Na_2_-EDTA, 10 *μ*M FeSO_4_·7H_2_O, 0.5 *μ*M MnSO_4_·H_2_O, 0.25 *μ*M ZnSO_4_·7H_2_O, 0.05 *μ*M CuSO_4_·5H_2_O, 100 *μ*M H_3_BO_3_, and 0.02 *μ*M (NH_4_)_6_Mo_7_O_24_. After that, seedlings were transferred to fresh Hoagland medium having two levels of Cd (applied as CdCl_2_·2.5 H_2_O), that is, 0 and 500 *μ*M. Seedlings were grown in the Cd stressful media for 24 hours. Next day, seedlings' roots were thoroughly washed with 20 mM EDTA-Na_2_ for 15 min to remove adhering metals. Then, seedlings were divided into roots, stems, and leaves for physiological, biochemical, and ultrastructural studies.

### 2.2. Measurements of Physiological Parameters

After 24-hour Cd stress, seedling roots, stems, and leaves were separated for the measurements of biomass-based tolerance indices and water contents. The fresh and dry biomass-based per plant tolerance indices and per plant water contents in roots, stems, and leaves were determined according to [4, 6], respectively. For each measurement, three replications were kept with different number of plants. Regarding fresh and dry biomasses, three plants per replication were taken.

### 2.3. Measurements of Photosynthesis and Light Harvesting Pigments

In order to evaluate leaf efficiency regarding its photosynthesis and chlorophyll fluorescence, method described by [14] was used. For the determination of chlorophyll pigments, 0.1 g FW per sample per replication was used. Leaves were first dark adapted for 15 min in order to measure all chlorophyll fluorescence parameters. Nonphotochemical quenching (NPQ) was measured using the protocol of [15]. And all measurements were taken from the same leaf. There were three replications and in each replication, three leaves were randomly selected from three different plants. And for every replication, the mean values were calculated for 15 different locations of the three different leaves.

### 2.4. Measurements of Cd Concentrations and Important Micro- and Macronutrients

For elemental analyses including Cd contents in roots, stem, and leaves, fifteen seedlings from each replicate were selected. At the end of the experiment, seedlings were washed three times first with tap and then with distilled water. To remove adhering metals from roots, they were immersed in 20 mM EDTA-Na_2_ for 15 min and were washed with dH_2_O for three-four times. Seedlings' roots, stems, and leaves were oven dried at 80°C for 48 hour. A 0.2 g of each sample was digested with a mixture of 5 mL HNO_3_ + 1 mL of HClO_4_, which was diluted to 25 mL using 2% HNO_3_ and then filtered. The concentrations of Cd and various micro- and macroelements in the filtrate were determined using inductively coupled plasma atomic emission spectroscope (ICP-AES, IRIS/AP optical emission spectrometer, Thermo Jarrel Ash, San Jose, CA) following standard procedures.

### 2.5. Quantification of MDA Contents, ROS, Total Soluble Proteins, and Antioxidants

Quantifications of oxidative stress markers such as MDA contents, hydrogen peroxide, superoxide radical (O_2_^•−^), extracellular hydroxyl radicals (OH^−^), total soluble proteins, and ROS-scavenging antioxidant activities were performed using established protocols described by [16]. A 0.5 g fresh sample of leaves, stems, and roots was used for all assays.

### 2.6. Ultramorphological and Microlocalization Studies

Cd-induced ultrastructural modifications in root meristem and leaf mesophyll cells were observed under transmission electron microscopy. Root and leaf samples were prepared according to [4, 6]. For scanning electron microscopy, leaf samples were first fixed with 2.5% glutaraldehyde in phosphate buffer (pH 7.0) for more than 4 hours and were washed three times with phosphate buffer for 15 min at each step. Then samples were postfixed with 1% OsO_4_ in phosphate buffer (pH 7.0) for 1 hour and washed three times with the same phosphate buffer for 15 min. The specimens were first dehydrated by a graded series of ethanol (50%, 70%, 80%, 90%, 95%, and 100%) for about 15 to 20 minutes at each step, transferred to the mixture of alcohol and iso-amyl acetate (v : v = 1 : 1) for about 30 minutes, and then transferred to pure iso-amyl acetate for about 1 hour. In the end, the specimens were dehydrated in Hitachi Model HCP-2 critical point dryer with liquid CO_2_. The dehydrated specimen was coated with gold-palladium and observed in Hitachi Model TM-1000 SEM.

For the Cd localization experiment, thin sections of 120 nm of both roots and leaves were prepared according to [4, 6]. They were observed in EDAX GENESIS XM2 30TEM energy spectrometer at 80 KV.

### 2.7. Statistical Analyses

The data obtained were subjected to one-way analysis of variance (ANOVA) using STATIX9. All the results are the means ± SE of three replications. Means were separated by Least Significant Difference (LSD) test at 5% level of significance.

## 3. Results and Discussion

Heavy metals-based pollution is a serious environmental threat for all living organisms. Cd is a highly phytotoxic heavy metal. Because of its water soluble nature [4], it is readily taken up by roots and transported to the vegetative and reproductive organs of plants. Resultantly, the mineral nutrition and homeostasis in plant shoot and root growth and developments [17] are greatly disturbed. Also, Cd can affect biochemical and structural aspects of cell by inducing oxidative stress and disruption of membrane composition and function [4, 6, 18].

### 3.1. Effect of Cd Stress on Tolerance Indices and Water Contents of Upland Cotton Seedlings

Biomass-based tolerance index is the indirect measurement of plant growth efficiency under stressful conditions. Tolerance index per plant based on both fresh and dry biomasses and water content percentage of roots, stems, and leaves of cotton seedlings is shown in Table 1. Mean data regarding tolerance index of both fresh and dry biomass revealed downward trends in roots, stems, and shoots at 500 *μ*M Cd as compared with the control. Greater and significant decline could be observed in tolerance index of dry biomass. As a whole, greater decline in tolerance index of roots followed by stem and leaves was noticed. Similar trend was observed by [19] in mustard cultivars under Cd stress. Cd-induced reduction in tolerance index directly reveals the growth inhibition of these parts. Inhibited growth may be due to Cd interference with the vital metabolic processes such as photosynthesis and translocation of photosynthetic products and essential nutrients [17]. That is why a general decline in the photosynthesis related parameters and essential nutrients was observed under Cd stress in the present experiments.

Measurement of water contents based on difference in fresh and dry biomass production is very helpful to investigate Cd-induced secondary stress, that is, water stress [6]. All parts of the seedlings absorbed more water at 500 *μ*M Cd as compared with the control. The root water content was significantly (*P* < 0.05) higher (4.67%) in comparison with leaves (1.42%) and stem (1.23%). As a whole, roots absorbed more water than leaves and stems, which are contrary to our previous findings [6] as well as those of [20] in pea and of [21] in *Lactuca *sp. This difference could be due to several reasons such as (a). We could not observe any wilting situation in cotton seedlings, (b). Ultramicroscopic observations revealed that most of the cells were in turgid conditions, (c). Upregulation of methionine synthase protein (as revealed by our proteomic studies, data not given here) might have caused greater lignification of the cell wall and more resistance to allow intracellular water out in Cd stressful conditions.

### 3.2. Effect of Cd on Photosynthetic Parameters of Cotton Seedlings

Photosynthesis is a major source of ROS production in plants, which performs active role in metabolism and formation of ROS [16]. Quantification of photosynthesis-related parameters gives a clear idea about the stressful effects of any external stimuli.  Table 2 shows various parameters of chlorophyll pigments and fluorescence. The mean data of chlorophyll pigments such as chlorophyll a, b and chlorophyll a/b ratio showed variable responses to Cd stress. The highest and statistically significant decline (52%) was observed only in chlorophyll a. Chlorophyll a and chlorophyll a/b ratio showed a decrease of 19% and 34%, respectively; however, this decrease was statistically nonsignificant. A similar trend in chlorophyll pigments composition was observed in *Brassica* under Cd stress [22].

The results for fluorescence parameters such as *F*_*m*_, *F*_*m*_′, and *F*_*v*_/*F*_*m*_ and nonquenching parameter (NPQ) reveal that Cd stress significantly inhibited the photosynthetic parameters with the exception of NPQ, which upregulated. Percent inhibition in the chlorophyll fluorescence parameters was in the order of *F*_*m*_ (33%) > *F*_*m*_′ (31%) >*F*_*v*_/*F*_*m*_ (20%). A decrease in the chlorophyll fluorescence was also observed in barley under Al stress [23] and in tomato under Cd stress [14]. Such reduction in chlorophyll pigments and fluorescence may lead to reduced photosynthesis and growth [24]. These inhibitory effects could be possibly due to indirect interaction of Cd with micronutrients (such Fe, Mn, Zn), which are made unavailable to act as cofactors of enzymes, pigments, and structural components of the photosynthetic apparatus [25]. Fe deficiency in leaves observed in the present experiments can be a responsible factor in Cd-induced inhibition of photosynthesis [26].

### 3.3. Analyses of Cd and Macro- and Micronutrients

There is a direct relationship of metal uptake in plants and its concentration in soil or medium [6].  Table 3 shows concentrations of Cd and various macro- and micronutrients in different parts of cotton seedlings grown for 24 hour in Cd stressed and nonstressed conditions. Under controlled conditions, Cd concentrations in roots, stems, and leaves of seedlings were almost negligible as compared to seedlings grown in 500 *μ*M of Cd, where all parts of the seedlings absorbed significant amounts of Cd. The highest Cd concentration (2.29 mg/g DW) was found in roots followed by stem (2.27 mg/g DW) and leaves (0.55 mg/g DW). Root retained more Cd and only a small portion was transported to aerial parts. Similar findings have been reported by [27].

Cadmium can interact with the availability of nutrients [28] and may imbalance the uptake and distribution of certain essential nutrients in plants [29]. In the present experiment, Cd stress had adverse effects on most of the macronutrients levels in roots, stems, and leaves as compared to their relevant controls (Table 3). Levels of macronutrients such as N, P, K, and Mg decreased in both roots and stems, while their levels enhanced in leaves at 500 *μ*M Cd as compared to their related controls. However, S level in all parts of cotton seedlings was upregulated. As a whole, maximum decrease was observed in K, which was in roots (75%), while significant enhancement was found in N in leaves (120%) as compared to their related controls. Furthermore, downregulation of macronutrients in roots was more than in stems. S contents levels showed a nonsignificant upward trend. Our present findings are contradictory to those of [20] in pea and [30] in birch.

Table 3 further depicts the micronutrients status in different parts of the cotton seedlings under Cd stress. The data reveal that Cd had a negative influence on the levels of various micronutrients except Fe and B. In roots of cotton seedlings, Fe contents nonsignificantly increased (21%) over the control, while the B contents levels in all parts of the cotton seedlings enhanced. Greater incline in B contents could be observed in roots, which was 235% as compared with the control, followed by stem (40%) and leaves (32%). As a whole the nutritional status of leaves and roots greatly altered. Almost similar trend was reported in pea [20]. Different factors like the involvement of different transporters in nutrients/elements translocation, variable affinity of phytochelatins for specific metals [20], and morphological changes of the conducting xylem tissues [31] could be the possible reasons for the differential uptake of these elements under Cd stress. Similar to our findings, Cd stress reduced the concentration of most of the nutrients in durum wheat [32] and barley [33].

### 3.4. Cd Stress Upregulated the MDA and ROS Contents and Downregulated the Total Soluble Protein Contents

MDA production is a cytotoxic product of lipid peroxidation [34], which is produced under stressful conditions. The production of superoxide radicals (O_2_^•−^), hydrogen peroxide (H_2_O_2_), and hydroxyl radicals (OH^−^) under Cd stress [16] has been reported. They can damage membrane and inactivate various enzymes due to reactions with proteins, lipids, and nucleic acids [35].

The main objective of the present study was to determine the effect of Cd stress on MDA contents and production of reactive oxygen species. Data in Table 4 reveal an increase in MDA and ROS in all parts of the cotton seedlings in response to Cd stress. Superoxide radical was produced in greater amount followed by H_2_O_2_, OH^−^, and MDA. Moreover, greater production of O_2_^•−^, H_2_O_2_, and MDA in leaves, while that OH^−^ in roots was found. Statistically significant variations were more in H_2_O_2_ and MDA at 5% probability level.

The total soluble proteins in all parts of the cotton seedlings were also determined in this study (Table 4). The tabulated data showed that in seedlings roots, protein contents were significantly higher (98%) in Cd treated seedlings, while in other parts it was lower (33% in stem and 37% in leaves), in comparison with the respective control. Similar upward trend in MDA and H_2_O_2_ was observed in *Brassica* under Cd stress [16, 22] and in wheat under heavy metal stress [36]. This increase in their production of these species indicates that Cd stress might have caused damage to membranes. This assumption is supported by the fractured plasma membrane, misshaped chloroplast, and enlarged vacuoles observed under Cd stress. Similar upregulation in MDA and ROS contents was observed in* Sedum alfredii* Hance [11] and *J. effuses* [18] under Cd stress. Such decline can be due to pigment loss, reduction in the photosynthetic efficiency, decreased RNA levels, and so forth. [37]. Increase in total soluble proteins in roots might be due to increase in number of nuclei, which might have synthesized greater amount of amino acids [4].

### 3.5. Effect of Cd Stress on Oxidative Metabolism Levels in Cotton Seedlings

Antioxidative enzymes play active roles in scavenging of ROS produced in plants under environmental stresses.  Table 5 shows the status of oxidative metabolism in different parts of cotton seedlings grown for 24 hours in Cd stressed and nonstressed conditions. Data regarding SOD activity showed an enhancement in its activity in all parts of the seedlings, which was statistically significant in roots and leaves. Greater percent enhancement was found in leaves (35%) followed by roots (18%) and stems (7%). Greater activity in leaves might be due the fact that Cd might have caused senescence-like situation in leaves. Such enhancement in SOD activity under Cd stress has also been previously reported in *Sedum alfredii* by [11]. Under Cd stress, the APX activity was reduced in different parts of the seedlings, but this effect was statistically not significant. Similar to our findings, a marked reduction in APX activity in roots of HE *Sedum alfredii* has been observed [11]. At higher Cd concentration (i.e., 500 *μ*M), activities of POD and GR were reduced by 34 and 85%, respectively, in roots, and the activity of CAT was reduced in stems by 83%. However, their activities were enhanced in all other parts of cotton seedlings. Greater relative increase in the activities of CAT, POD, and GR was observed in leaves (169, 58%) and stems (245%), respectively. However, POD activity enhanced in leaves under Cd stress. Our present findings are not in line with those of [20]. Its activity was also increased in *B. juncea* under Cd stress [38]. As a whole, significant percent inhibition (85%) was in GR activity of roots, while significant percent enhancement (245%) was also found in the GR activity of stem. Such increase has also been found in mustard under cadmium stress [37].

### 3.6. Effect of Cd Stress on Ultrastructure of Roots and Leaves

**Figure 1:**
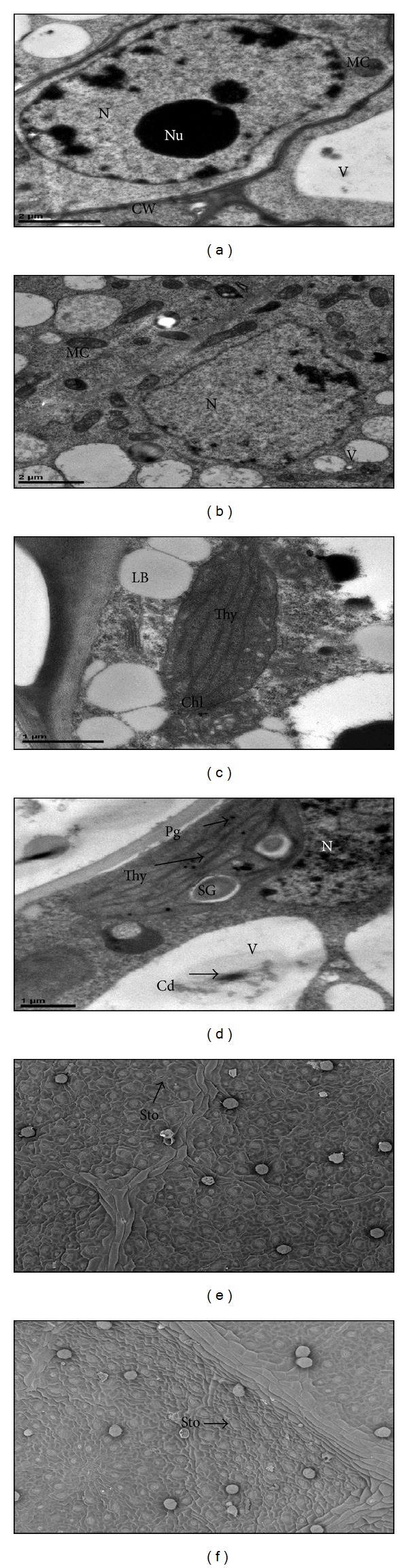
Transmission electron micrographs of roots meristem cells ((a), (b)), leaf mesophyll cells ((c), (d)), and scanning micrographs EM of leaves ((e), (f)) of cotton seedlings under normal ((a), (c), (e)) and Cd stress ((b), (d), (f)) conditions. MC: mitochondria, CW: cell wall, N: nucleus, Nu: nucleolus, V: vacuole, Chl: chloroplast, Thy: thylakoids, LB: lipid bodies, Pg: plastoglobuli, SG: starch granules, Sto: stomata.

Ultrastructural studies in plants are important tools to peep into the cellular mechanisms being involved in the detoxification of Cd. The ultrastructural changes, in combination with metabolic activities, help devise a strategy to reduce the effects of Cd stress in plants. Ultrastructural alterations in root meristem and leaf mesophyll cells of ZMS-49 were not so severe at 500 *μ*M Cd as compared with the control (Figures 1(a)–1(d)). At 0 *μ*M Cd, the cells of root meristems had typical structural features. They possessed granular cytoplasm with a number of vacuoles, mitochondria, and endoplasmic reticulum. Membranous structures such as plasma, nucleus and mitochondria were smooth. Cytoplasm was dense with centrally located nucleus (Figure 1(a)). At 500 *μ*M Cd level, ultrastructural changes such as increase in number of vacuoles, nucleoli, mitochondria, misshaped nucleus, and fractured nuclear membrane were observed. However, plasmolysis was almost absent and electron dense precipitates, probably Cd, were observed in vacuoles and intracellular spaces as well as attached to the cell walls (Figure 1(b)).

The transmission electron microscopy images of leaf mesophyll cells are shown in Figures 1(c) and 1(d). Under normal conditions, thin and clean cell walls were seen, with well-shaped nucleus and few lipid bodies. The chloroplasts were of regular shape with well-arranged thylakoids (Figure 1(c)). However, some alterations were observed at whole leaf mesophyll as well as chloroplast levels. Greater modifications could be seen in vacuolar, nuclear, and chloroplastic regions. An increase in number of lipid bodies, starch granules, and plastoglobuli could be noticed (Figure 1(d)). Electron dense precipitates, probably Cd, were mostly seen in the vacuolar and cell walls regions. Such observations have also been made in previous studies [4, 6, 16, 18, 22]. Increase in number of starch granules is a general sign of stress in plants [18]. Increased nutrient deficiency or disturbed vein loading system [6] due to high Cd translocation into shoot may lead to starch accumulation in the chloroplast. Their deposition in these regions shows that ZMS-49 can play a significant role in Cd tolerance by preventing the circulation of free Cd ions in the cytosol [4]. Our findings are further supported by [39, 40].

Figures 1(e) and 1(f) also show the scanning micrographs of the abaxial side of cotton leaf. These micrographs show that almost outer surfaces were smooth in both Cd stressed and nonstressed leaves of cotton seedlings. Less number of stomata was found closed in the Cd treated leaf mesophyll cells. The number of stomata was almost the same in both types of cells. Trichome was less turgid in the Cd treated leaf mesophyll cells as compared with the nonstressed leaves. Such observations are against those of [20]. Taking together observations at transmission and scanning microscopic levels, it is argued that Cd stress caused very little alterations in both roots and leaves. There was observed some senescent-like situation in cellular compartments of the cells of these parts, mostly in leaves, which might be due to increased production of MDA and various ROS.

### 3.7. Microlocalization of Cd

**Figure 2:**
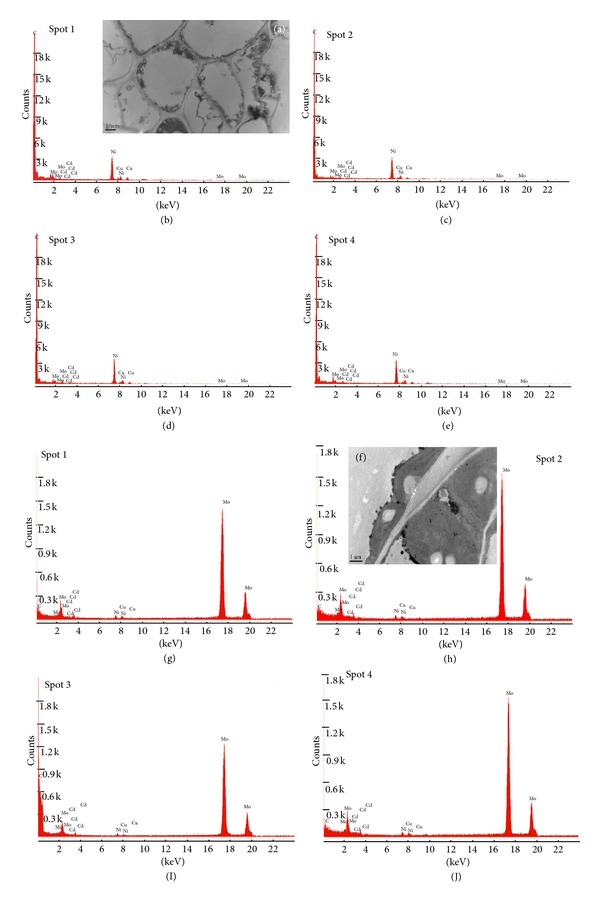
Energy dispersive X-ray analysis of both roots ((a)–(e)) and leaves ((f)–(j)) under Cd stress. Peaks show the presence of Cd in vacuoles and cell walls of these samples.

Compartmentalization and complexation of heavy metals at a subcellular level play an important role in detoxification of heavy metals in plant tissues [41]. In our present experiment, we found electron dense precipitates in the Cd treated root meristems and leaf mesophyll cells (Figures 2(a) and 2(f)). These were confirmed by EDX technology. For every root and leaf samples, we analyzed four different spots. The EDX spectra obtained confirmed the presence of Cd mostly in the dead parts of the cell such as cell wall, vacuoles, and intracellular spaces (Figures 2(b)–2(e) and 2(g)–2(j)). We only observed the Cd treated samples of roots and leaves for the microlocalization of Cd because such precipitates were not clearly observed in the control samples. Such observations have also been previously made by [7].

## 4. Conclusions

From the present study, it can be concluded that

- Cd stress disturbed photosynthetic machinery and nutrient levels, which indirectly reduced biomass-based tolerance index;
- there was an increase in the water contents in different parts of the cotton seedlings in order to combat such stressful situation;
- there was a rise in MDA and ROS contents in all parts, which were scavenged by various ROS-scavenging antioxidants due to their upregulation;
- the active involvement of ROS-scavenging antioxidant machinery caused less disruption of cellular organelles both in roots and leaves as well as Cd deposition in cell wall and vacuole.

## Figures and Tables

**Table 1:** Tolerance indices/plant based on biomass (fresh and dry) and water content (%)/plant of different parts of cotton seedlings grown under Cd stress.

Parts	Tolerance index (FW)		Tolerance index (DW)		Water contents (%)	
	0 *µ*M Cd	500 *µ*M Cd	0 *µ*M Cd	500 *µ*M Cd	0 *µ*M Cd	500 *µ*M Cd
Roots	100.00 ± 0.00^a^ (0.00)	62.86 ± 2.44^b^ (−37.14)	100.00 ± 0.00^a^ (0.00)	26.11 ± 3.89^b^ (−73.89)	92.47 ± 0.29^b^ (0.00)	96.78 ± 0.73^a^ (4.67)
Stems	100.00 ± 0.00^a^ (0.00)	74.58 ± 6.73^b^ (−25.42)	100.00 ± 0.00^a^ (0.00)	65.00 ± 5.00^b^ (−35.00)	91.10 ± 0.22^b^ (0.00)	92.22 ± 0.29^a^ (1.23)
Leaves	100.00 ± 0.00^a^ (0.00)	99.33 ± 9.66^a^ (−0.97)	100.00 ± 0.00^a^ (0.00)	85.39 ± 2.56^b^ (−14.61)	90.22 ± 0.85^a^ (0.00)	91.50 ± 0.64^a^ (1.42)

Values are the means ± SE of three replications. Variants possessing the same letter are not statistically significant at *P* < 0.05. Values in parenthesis show percent relative increase (+) or decrease (−) over the related controls.

**Table 2:** Chlorophyll pigments and fluorescence of leaves of cotton seedlings grown under Cd stress.

Cd levels	Chlorophyll pigments			Chlorophyll fluorescence			
	Chl a	Chl b	Chl a/b	*F*_*m*_	*F*_*m*_′	*F*_*v*_/*F*_*m*_	NPQ (*F*_*m*_/*F*_*m*_′ − 1)
0 *µ*M	0.044 ± 0.00^a^ (0.00)	2.72 ± 0.13^a^ (0.00)	0.016 ± 0.003^a^ (0.00)	0.49 ± 0.05^a^ (0.00)	0.49 ± 0.01^a^ (0.00)	0.81 ± 0.02^a^ (0.00)	0.03 ± 0.08^a^ (0.00)
500 *µ*M	0.021 ± 0.01^b^ (−52.27)	2.22 ± 0.27^a^ (−19.12)	0.011 ± 0.005^a^ (−33.83)	0.33 ± 0.03^b^ (−32.65)	0.34 ± 0.01^b^ (−30.61)	0.65 ± 0.02^b^ (−19.75)	0.05 ± 0.03^a^ (55.17)

Values are the means ± SE of three replications. Variants possessing the same letter are not statistically significant at *P* < 0.05. Values in parenthesis show percent relative increase (+) or decrease (−) over the related controls.

**Table 3:** Cd uptake by different parts of cotton seedlings and macro- and micronutrients concentration in different parts of the cotton seedlings under Cd stress.

Elements (mg/g DW)	Roots		Stems		Leaves	
	0 *µ*M Cd	500 *µ*M Cd	0 *µ*M Cd	500 *µ*M Cd	0 *µ*M Cd	500 *µ*M Cd
Cd	0.04 ± 0.004^b^ (0.00)	2.29 ± 0.07^a^ (6206.61)	0.004 ± 0.001^b^ (0.00)	2.27 ± 0.02^a^ (55561.05)	0.002 ± 0.003^b^ (0.00)	0.55 ± 0.04^a^ (30798.29)

Macronutrients						
N	19.51 ± 0.83^a^ (0.00)	9.78 ± 1.40^b^ (−49.84)	5.24 ± 0.58^a^ (0.00)	3.15 ± 0.97^a^ (−39.84)	1.11 ± 0.23^b^ (0.00)	2.45 ± 0.73^a^ (120.28)
P	5.51 ± 0.94^a^ (0.00)	2.80 ± 0.56^a^ (−49.12)	9.06 ± 1.52^a^ (0.00)	7.73 ± 0.41^a^ (−14.68)	10.68 ± 0.96^a^ (0.00)	12.95 ± 0.91^a^ (21.29)
K	33.29 ± 1.63^a^ (0.00)	8.30 ± 0.75^b^ (−75.06)	27.59 ± 1.32^a^ (0.00)	23.32 ± 1.41^a^ (−15.51)	18.70 ± 1.45^a^ (0.00)	21.65 ± 1.56^a^ (15.77)
Mg	8.48 ± 0.35^b^ (0.00)	4.67 ± 0.56^a^ (−44.88)	5.73 ± 0.39^a^ (0.00)	5.24 ± 0.47^a^ (−8.58)	5.19 ± 0.79^a^ (0.00)	6.24 ± 0.28^a^ (20.35)
S	15.68 ± 0.92^a^ (0.00)	19.59 ± 1.79^a^ (25.00)	7.66 ± 0.69^a^ (0.00)	12.32 ± 1.73^a^ (60.74)	9.76 ± 0.43^a^ (0.00)	11.62 ± 0.76^a^ (18.98)

Micronutrients						
Fe	0.94 ± 0.12^a^ (0.00)	1.14 ± 0.13^a^ (21.15)	0.06 ± 0.01^a^ (0.00)	0.05 ± 0.001^a^ (−21.67)	0.15 ± 0.005^a^ (0.00)	0.12 ± 0.003^b^ (−21.68)
Zn	0.29 ± 0.004^a^ (0.00)	0.13 ± 0.01^b^ (−54.91)	0.08 ± 0.006^a^ (0.00)	0.06 ± 0.004^a^ (−19.35)	0.10 ± 0.004^a^ (0.00)	0.080 ± 0.006^a^ (−16.61)
Cu	0.02 ± 0.001^a^ (0.00)	0.02 ± 0.001^b^ (−33.33)	0.01 ± 0.001^a^ (0.00)	0.01 ± 0.000^b^ (−23.17)	0.01 ± 0.000^a^ (0.00)	0.01 ± 0.001^a^ (−8.28)
Ca	7.65 ± 0.24^a^ (0.00)	4.20 ± 0.4^b^ (−45.09)	20.52 ± 0.81^a^ (0.00)	16.97 ± 0.35^b^ (−17.29)	21.04 ± 0.79^a^ (0.00)	19.90 ± 0.65^a^ (−5.51)
Mn	54.81 ± 0.004^a^ (0.00)	31.52 ± 0.003^b^ (−42.50)	19.07 ± 0.003^a^ (0.00)	17.26 ± 0.001^a^ (−9.50)	93.97 ± 0.003^a^ (0.00)	83.94 ± 0.002^b^ (−10.67)
Ni	0.01 ± 0.001^a^ (0.00)	0.01 ± 0.001^a^ (−36.05)	0.02 ± 0.000^a^ (0.00)	0.002 ± 0.000^a^ (−13.07)	0.002 ± 0.000^a^ (0.00)	0.001 ± 0.000^a^ (−16.25)
B	0.04 ± 0.000^a^ (0.00)	0.05 ± 0.000^a^ (234.54)	0.01 ± 0.004^a^ (0.00)	0.01 ± 0.002^a^ (40.04)	0.04 ± 0.002^a^ (0.00)	0.050 ± 0.004^a^ (32.40)

Values are the means ± SE of three replications. Variants possessing the same letter are not statistically significant at *P* < 0.05. Values in parenthesis show percent relative increase (+) or decrease (−) over the related controls.

**Table 4:** Effect of Cd stress on MDA (*η*M/mg protein) and ROS (H_2_O_2_, O_2_^•−^, OH^•^) contents (*μ*M/gFW) as well as total soluble proteins (mg/gFW) in different parts of cotton seedlings.

Traits	Parts					
	Roots		Stems		Leaves	
	0 *µ*M Cd	500 *µ*M Cd	0 *µ*M Cd	500 *µ*M Cd	0 *µ*M Cd	500 *µ*M Cd
MDA	9.18 ± 0.24^b^ (0.00)	11.25 ± 0.23^a^ (22.55)	10.83 ± 0.24^a^ (0.00)	11.73 ± 0.27^a^ (8.47)	13.14 ± 21.61^b^ (0.00)	21.61 ± 2.24^a^ (64.46)
H_2_O_2_	20.92 ± 6.21^a^ (0.00)	34.17 ± 12.34^a^ (63.30)	42.44 ± 9.91^b^ (0.00)	80.69 ± 8.37^a^ (90.13)	68.18 ± 10.09^b^ (0.00)	164.16 ± 26.16^a^ (140.77)
O_2_^•−^	22.15 ± 9.00^a^ (0.00)	33.66 ± 22.85^a^ (51.95)	75.23 ± 36.76^a^ (0.00)	102.78 ± 42.28^a^ (36.62)	19.29 ± 1.09^b^ (0.00)	126.96 ± 6.18^a^ (558.16)
OH^•^	0.10 ± 0.01^b^ (0.00)	0.15 ± 1.53^a^ (103.36)	0.13 ± 0.03^a^ (0.00)	0.19 ± 0.03^a^ (52.72)	0.13 ± 0.08^a^ (0.00)	0.15 ± 0.08^a^ (13.83)
Proteins	8.89 ± 0.19^b^ (0.00)	17.63 ± 0.86^a^ (98.34)	13.06 ± 4.31^a^ (0.00)	8.74 ± 0.17^a^ (−33.08)	14.81 ± 1.71^a^ (0.00)	9.33 ± 0.65^b^ (−36.99)

Values are the means ± SE of three replications. Variants possessing the same letter are not statistically significant at *P* < 0.05. Values in parenthesis show percent relative increase (+) or decrease (−) over the related controls.

**Table 5:** Antioxidants status in different parts of cotton seedlings upon their exposure to Cd stress for 24-hour duration.

Antioxidants	Parts					
	Roots		Stems		Leaves	
	0 *µ*M Cd	500 *µ*M Cd	0 *µ*M Cd	500 *µ*M Cd	0 *µ*M Cd	500 *µ*M Cd
SOD (U/mg protein)	275.25 ± 1.69^b^ (0.00)	324.67 ± 6.13^a^ (17.95)	267.13 ± 8.21^a^ (0.00)	284.50 ± 1.94^a^ (6.50)	255.82 ± 21.26^b^ (0.00)	344.92 ± 5.09^a^ (34.83)
APX (*µ*M/min/mg protein)	0.51 ± 0.17^a^ (0.00)	0.41 ± 0.24^a^ (−20.04)	1.43 ± 0.95^a^ (0.00)	0.37 ± 0.10^a^ (−74.50)	3.31 ± 0.16^a^ (0.00)	1.84 ± 0.35^a^ (−44.43)
CAT (*µ*M/min/mg protein)	0.030 ± 0.02^a^ (0.00)	0.035 ± 0.01^a^ (12.37)	0.11 ± 0.02^a^ (0.00)	0.02 ± 0.010^b^ (−83.17)	0.21 ± 0.010^a^ (0.00)	0.57 ± 0.02^a^ (169.09)
POD (*µ*M/min/mg protein)	7.48 ± 0.69^a^ (0.00)	4.93 ± 0.33^b^ (−34.09)	2.62 ± 0.13^b^ (0.00)	3.72 ± 0.15^a^ (41.85)	5.07 ± 0.14^b^ (0.00)	8.03 ± 0.39^a^ (58.26)
GR (*µ*M/min/mg protein)	0.22 ± 0.03^a^ (0.00)	0.03 ± 0.08^a^ (−85.10)	0.05 ± 0.02^b^ (0.00)	0.18 ± 0.02^a^ (244.76)	0.17 ± 0.02^b^ (0.00)	0.27 ± 0.03^a^ (54.59)

Values are the means ± SE of three replications. Variants possessing the same letter are not statistically significant at *P* < 0.05. Values in parenthesis show percent relative increase (+) or decrease (−) over the related controls.

## Abbreviations

APX: Ascorbate peroxidase
CAT: Catalase
MDA: Malondialdehyde
POD: Peroxidase
ROS: Reactive oxygen species
SOD: Superoxide dismutase
H_2_O_2_: Hydrogen peroxide.
